# Pathogenesis and Clinical Management of Metabolic Dysfunction-Associated Steatotic Liver Disease

**DOI:** 10.3390/ijms26125717

**Published:** 2025-06-14

**Authors:** Roxana Liana Lucaciu, Sorina Cezara Coste, Adriana Corina Hangan, Mihaela Iancu, Olga Hilda Orășan, Angela Cozma, Sidonia Gog Bogdan, Lucia Maria Procopciuc

**Affiliations:** 1 Department of Pharmaceutical Biochemistry and Clinical Laboratory, Faculty of Pharmacy, “Iuliu Hațieganu” University of Medicine and Pharmacy, 400012 Cluj-Napoca, Romania; liana.lucaciu@umfcluj.ro; 2 4th Department of Internal Medicine, Faculty of Medicine, “Iuliu Hațieganu” University of Medicine and Pharmacy, 400012 Cluj-Napoca, Romania; secara.sorina@umfcluj.ro (S.C.C.); hilda.orasan@umfcluj.ro (O.H.O.); angelacozma@umfcluj.ro (A.C.); 3 Department of Inorganic Chemistry, Faculty of Pharmacy, “Iuliu Hațieganu” University of Medicine and Pharmacy, 400012 Cluj-Napoca, Romania; adriana.hangan@umfcluj.ro; 4 Medical Informatics and Biostatistics, Faculty of Nursing and Health Science, “Iuliu Hațieganu” University of Medicine and Pharmacy, 400012 Cluj-Napoca, Romania; miancu@umfcluj.ro; 5 Department of Sugery and ATI, Faculty of Veterinary Medicine, University of Agricultural Sciences and Veterinary Medicine, 400372 Cluj-Napoca, Romania; sidonia.bogdan@usamvcluj.ro; 6 Medical Biochemistry, Department of Molecular Sciences, Faculty of Medicine, “Iuliu Hațieganu” University of Medicine and Pharmacy, 400349 Cluj-Napoca, Romania

**Keywords:** MASLD, NAFLD, pathogenesis, diagnosis, lifestyle, therapies

## Abstract

Metabolic dysfunction-associated steatotic liver disease (MASLD) is a chronic progressive liver disease with a substantial impact on global health. Given that MASLD has a complex etiology, it is a multisystemic disease, a multidisciplinary approach is required when treating MASLD. The optimal drug for MASLD should diminish steatosis, fibrosis and inflammation in the liver. Although the pharmaceutical industry is still lagging in developing an approved pharmacologic therapy for MASLD, research has recently intensified, and many molecules that are in the final stages of clinical trials are expected to be approved in the coming few years. The current review updated information related to the MASLD pathogenesis, diagnosis and therapeutic options, how patients are clinically managed nowadays, and what to expect in the near future.

## 1. Introduction

Non-alcoholic fatty liver disease (NAFLD) is characterized by the excessive deposition of triglycerides in hepatocytes, and is considered to be a metabolic liver disease [1]. It represents a generic name for several liver diseases, which include non-alcoholic fatty liver (NAFL), simple fatty liver (SFL), non-alcoholic steatohepatitis (NASH), cirrhosis, and hepatocellular carcinoma (HCC) [2]. Nowadays, the research concerning NAFLD has become urgent and important because the age of diagnosis of NAFLD is progressively decreasing, and its relationship with cancer risk is well known [3].

After understanding the pathogenesis of NAFLD, NAFLD became MASLD (metabolic dysfunction-associated steatotic liver disease), non-alcoholic fatty liver (NAFL) became MAFL (metabolic-associated fatty liver), and NASH became MASH (metabolic dysfunction-associated steatohepatitis) [4]. In this way, MASLD became a prominent global health challenge [5]. It is characterised by the excess accumulation of hepatic lipids (hepatic steatosis), which can lead to inflammation (steatohepatitis) and progressive fibrosis.

The clinical evolution of MASLD is unpredictable. In unfavorable cases, MASLD can be fatal. In unfavorable cases, MASLD can progress to advanced liver diseases such as cirrhosis, hepatic decompensation, or hepatocellular carcinoma, which significantly increase the risk of liver-related mortality. Additionally, MASLD is often associated with increased cardiovascular risk, which can also contribute to fatal outcomes. In general, MASLD remains asymptomatic until decompensated cirrhosis develops. When symptoms appear, they consist of a mild pain in the right upper quadrant and in fatigue [6].

The severity of MASLD is mediated by factors that include genetic susceptibility, nutritional content, adiposity, insulin resistance, the gut microbiome, and a host of endocrine effectors of disease [7]. The pathogenesis and the clinical manifestations of MASLD are heterogenous because in MASLD are involved many and different pathogenic molecular pathways [8,9]. Obesity and adipose tissue dysfunction (adiposopathy) are major risk factors for MASLD [10]. These conditions are closely associated with metabolic disturbances such as insulin resistance, type 2 diabetes mellitus (T2DM), hypertension, and dyslipidemia, which are commonly observed in patients with MASLD [11].

As a result of the interactions between genetic, epigenetic and environmental factors the pathogenesis of MASH/MASLD is complex and complicated at the same time. The consequence of these interactions are represented by systemic metabolic dysfunction involving the liver, with deranged molecular pathways and cell-cell communication between hepatocytes, liver sinusoidal cells, stellate cells, Kupffer cells, and recruited immune cells [12]. The variable combinations of these factors can generate different clinical phenotypes among individuals with MASLD. This is the reason why it is necessary to develop a personalized management of the disease.

MASLD itself determines the occurrence of some extrahepatic complications like cardiovascular disease, T2DM/insulin-resistance, chronic kidney disease, and a few types of extrahepatic cancers [13,14]. Patients with severe liver fibrosis likely develop subclinical carotid atherosclerosis with cardiovascular diseases, which represent the leading cause of MASLD-related mortality [15].

Death rates from extrahepatic cancers in MASLD exceed the deaths caused by hepatocellular carcinoma because the global prevalence of MASLD is about 30%. It was found that the increased extrahepatic cancer risk in MASLD is independent of the liver fibrosis stage. Taking into account this significant prevalence, in the future, access to care and the costs for treating MASLD will be huge [16].

The diagnosis of MASLD is increasingly moving toward non-invasive, early detection methods due to its often asymptomatic nature and the rising global burden. Traditional reliance on liver biopsy, while accurate, is impractical for widespread use, particularly in lean MASLD, which defies typical obesity-associated expectations and complicates diagnosis with limited awareness and outdated criteria [17]. FibroScan liver elastography has emerged as a rapid and effective diagnostic tool capable of detecting hepatic steatosis and fibrosis in early and subclinical stages, proving valuable in adult and pediatric populations [18]. Imaging-based modalities like MRI and ultrasound have demonstrated promising diagnostic accuracy, with AUROC values as high as 0.94–1.0, however, inconsistent pathology definitions and a lack of validation across studies hinder standardization [19]. Meanwhile, extracellular vesicles (EVs) offer novel diagnostic and therapeutic potential due to their role in the pathophysiological alterations of liver tissue, positioning them as next-generation biomarkers under active investigation [20]. Collectively, these advancements represent a significant evolution in MASLD diagnostics, shifting from invasive procedures to integrated, non-invasive tools that emphasize early detection, personalized risk profiling, and continuous monitoring.

Multidisciplinary teams composed of hepatologists, cardiologists, endocrinologists, physiotherapists, dietitians, and psychologists must be involved in treating patients with MASLD [21]. The optimal treatment of MASLD must also involve the metabolic risk factors that promote cancer and cardiovascular disease, not only the progression of liver disease. Considering that MASLD is a disease with slow evolution, clinical endpoints are difficult to achieve and assess in clinical trials. Within them is followed, in the first place, the fibrosis reversal, since fibrosis severity is the main prognostic factor in MASLD. The biomarkers involved in MASLD and its molecular mechanisms are not completely understood, so finding an appropriate pharmacological treatment represents a challenge [22]. Optimal treatment of MASLD must have a safety profile, in order not to cause more harm than good to some asymptomatic patients, who may be morbid event-free for many years [23].

The current review presents information regarding MASLD pathogenesis, diagnosis and therapeutic options, how patients are clinically managed nowadays, and what to expect in the near future.

## 2. MASLD Pathogenesis

### 2.1. Physiopathology of Liver Fibrosis

**Figure 1:**
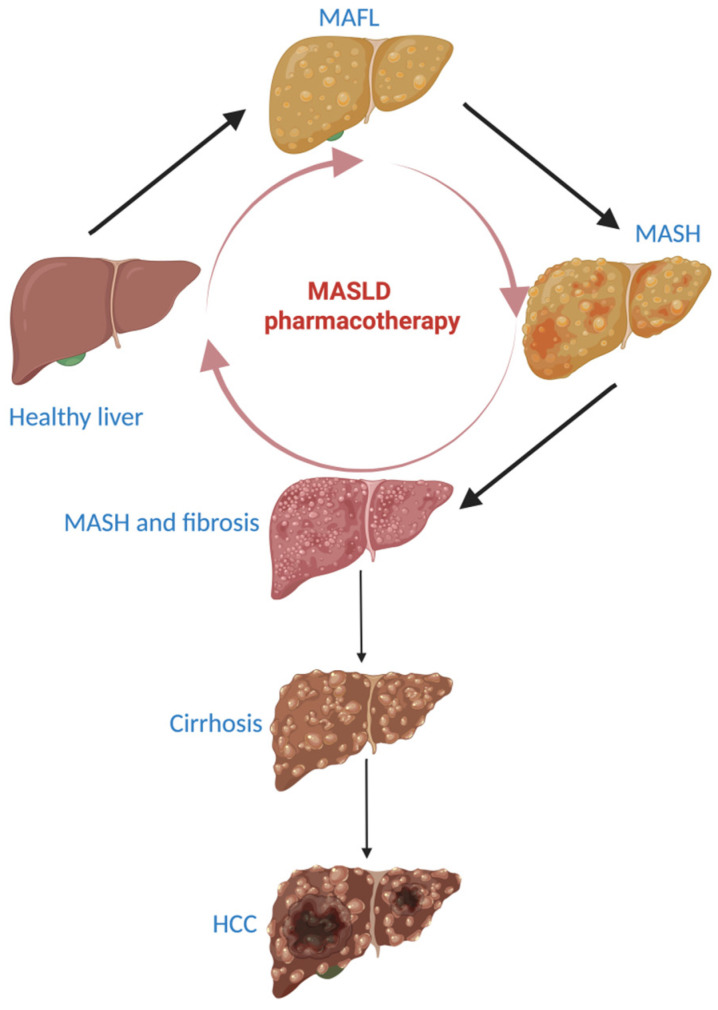
Exacerbation of MASLD and pharmacotherapy to reverse the stage of fibrotic MASH to a healthy liver. MASLD: metabolic dysfunction-associated steatotic liver disease, MAFL: metabolic-associated fatty liver, MASH: metabolic-associated steatohepatitis, HCC: hepatocellular carcinoma. Dark arrow: worsening liver condition. Light red arrow: improving MASLD stages under MASLD pharmacotherapy.

Insulin promotes the storage of free fatty acids (FFAs) and, consequently, of triglycerides at the adipose tissue level. This activity is due to its anti-lipolytic activity in lipid metabolism. If the insulin resistance is present, insulin isn’t able to inhibit adipocyte lipolysis and this determines the release of FFAs from the adipose tissue in the bloodstream and consequently their excessive presence in the liver, where an excessive biosynthesis of triglycerides takes place [24]. One of the leading causes of fatty liver is represented by insulin resistance and this also represents one of the therapeutic targets in MASLD treatment [25]. The source of FFAs from the liver is represented by the diet, de novo lipogenesis and adipose tissue (60%). Excessive accumulation of FFAs in the liver is considered to be responsible for the pathogenic pathway of liver fibrosis and the formation of reactive oxygen species [26]. The excess of FFA in the liver generates lipotoxic lipids that cause endoplasmic reticulum stress, oxidative stress, inflammation, and apoptosis of hepatocytes. All of these are involved in the formation of reactive oxygen species. In this environment, Kupffer cells (macrophages in the liver sinusoids) release the profibrotic factor TGF-β (transforming growth factor-β), which activates the hepatic stellate cells. The activated hepatic stellate cells migrate to the place of the injury, secrete an extracellular matrix and determine the formation of a fibrotic tissue in the liver. If fibrinogenesis is exacerbated, it becomes pathogenic [27,28]. The severity of liver fibrosis reflects the liver damage. An extensive liver fibrosis indicates the presence of compensated or uncompensated cirrhosis and of HCC (Figure 1) [29,30].

### 2.2. MASLD and the Disruption of Carbohydrate Metabolism

In the onset and progression of MASLD/MASH, glucose metabolism has an essential role [31]. MASLD patients have increased levels of hexokinase 2 (HK2) and pyruvate kinase isozyme type M2 (PKM2), two essential enzymes involved in glycolysis. The increased level of the two enzymes activates the glycolytic process. As a consequence of an increase in glucose transport to the liver, glycolysis will also be increased. So, a large amount of pyruvate will be available for “de novo” lipogenesis. MASLD is characterized by an abnormal accumulation of triglycerides in hepatocytes [32]. The formation of lactate represents the alternative pathway of pyruvate metabolism. Lactate is involved in decreasing the activity of histone deacetylase (HDAC), which represents a step in stimulating the “de novo” lipogenesis pathway. The uptake of FFAs by hepatocytes and the promotion of the expression of lipogenic genes are stimulated by the increased levels of lactic acid. Mitochondrial dysfunction and a deranged Krebs cycle lead to oxidative stress and DNA damage in the case of steatohepatitis [33].

Insulin resistance may be hepatic or systemic. Both have a negative influence on MASLD. After a short-term consumption of high-fat diets, hepatic insulin resistance can be triggered. It is not accompanied by peripheral insulin resistance. The insulin resistance is involved in increasing gluconeogenesis and “de novo” lipogenesis [34]. To increase the “de novo” lipogenesis, insulin participates in the activation of the liver X receptor (LXR), with upregulation of CHREBP1 and SREBP1 genes [35]. Insulin is also involved in the regulation of very low-density lipoprotein (VLDL) production because it inhibits the microsomal triglyceride transport protein (MTTP) and promotes the apolipoprotein B (ApoB) degradation. With the emergence of insulin resistance, problems appear in the regulation of VLDL due to the reversal of the above-mentioned processes, to an increased uptake of FFAs in the liver and to a decrease in the phosphorylation of forkhead box transcription factor 1 (FoxO1). All these are involved in MASLD pathogenesis [36]”.

Fructose is another carbohydrate which can worsen hepatic steatosis. It has important roles in maintaining metabolic homeostasis. In the liver, the catabolism of fructose in the aim to generate substrates involved in the “de novo” lipogenesis pathway takes place in the presence of phosphofructokinase [37]. While fructose stimulates the “de novo” lipogenesis pathway it inhibits the β-oxidation process. It is also involved in decreasing the inhibition of protein biosynthesis and in increasing oxidative stress. All these worsen hepatic steatosis [38].

### 2.3. MASLD and the Disruption of Lipid Metabolism

In MASLD, hepatocellular lipid accumulation, mainly as triglycerides, appears as a consequence of the imbalance between lipid input and output at this level. For triglycerides synthesis FFAs represent the main substrate [39]. An excessive accumulation of FFAs in the liver causes a cascade of harmful events. These include lipotoxicity [40], the dysfunction of mitochondrial and endoplasmic reticulum, the activation of signaling pathways related to inflammation and metabolism, and the activation of the receptor involved in inflammation [41]. FFAs and diacylglycerols (intermediates in de novo lipogenesis) are involved in the disruption of metabolic homeostasis having as a result an excessive production of reactive oxygen species (ROS), which originate from impaired mitochondrial function [42]. At least initially, FFAs are esterified and transported via VLDL and/or oxidized with conversion to different substrates. This initial defense mechanism fails if FFAs exceed the mitochondria’s capacity to metabolize FFAs, leading to increased ROS production. This determines the evolution from steatosis to MASH [43].

Lipolysis begins with the production of cyclic adenosine monophosphate (cAMP). cAMP activates protein kinase A, which in turn activates specific lipases, namely phosphohormone-sensitive lipase (p-HSL) and phospho-perilipin 1 (p-PLIN1), by phosphorylation. After lipolysis takes place, free FFAs are directed to the liver. Insulin is known to inhibit these pathways [44].

In MASLD, these mechanisms are upregulated in the adipose tissue and are independent to the diabetes presence. Due to the adipocyte hypertrophy and insulin resistance, obesity can occur. Lipolysis will increase and consecutive, the uptake of FFAs into the liver is exacerbated [34].

The gut microbiota is involved in regulating systemic homeostasis. This role is partly dependent on bile acids (BA) metabolism-mediated signal transduction together with specific receptors [45]. The damage observed in chronic liver diseases, which also includes the evolution from steatosis to steatohepatitis, is also due to the alteration of the BAs’ function and disruption of their metabolism. This occurs independently of the presence of obesity and diabetes [46].

The enterohepatic circulation of BAs is partially interrupted by the inhibition of the ileal/colonic reabsorption and increases the fecal excretion of BAs. Consequently, a greater amount of cholesterol is metabolized into bile acids, thus reducing the risk of obesity. An important relevance is that BAs serve as ligands for Farnesoid X receptor agonists (FXR) during their absorption in the terminal ileum, acting as a hormone [47].

In the liver FXR fulfills several roles namely, inhibits lipogenesis pathway by inhibiting SREBP1c, promotes the oxidation of FFAs and promotes ketogenesis, processes that are related to fibroblast growth factor 21 (FGF21) [48] and induces β-oxidation by activating peroxisome proliferator-activated receptor-α (PPARα). In the blood stream FXR facilitates the clearance of VLDL, improving metabolic dysfunction in MASLD [49].

After FXR intestinal activation, the intestinal epithelial cells release human fibroblast growth factor 15/19 (FGF15/19) into the liver. This reduces hepatic steatosis and increases insulin resistance [50]. Also, FXR intestinal activation decreases glucagon-like peptide-1 (GLP-1) secretion. Because FXR is distributed in several tissues, the impact of FXR on MASLD is still being studied [51].

### 2.4. MASLD and Thyroid Hormones

Thyroid hormones acting on the thyroid hormone receptor isoforms α (THRα) receptor stimulate hepatic lipogenesis while THRβ appears to play a larger role in fatty acid oxidation. THR-β is also known as nuclear receptor subfamily 1, group A, member 2 (NR1A2), a nuclear receptor protein encoded by the THRβ gene in humans. It represents a sensor for triiodothyronine and is involved in liver regeneration and reduction of apoptosis at this level. It also improves hepatic regulation of lipid metabolism and plays a role in regulating insulin sensitivity [52].

The liver not only receives signals from thyroid hormones, but also has receptors for thyroid-stimulating hormone (TSH, or thyrotropin), which induce hepatic steatosis, and is a major site for the conversion of thyroxine (T4) to triiodothyronine (T3). It also synthesizes the main proteins to which thyroid hormones bind: thyroxine-binding globulin, transthyretin, and albumin [7,53].

Thyroid hormones stimulate THR-β. THR-β is mostly expressed in the liver. Consequently, the lipid metabolism in the liver is accelerated as well as the mobilization of the FFA at this level. As a result, there is a reduction in LDL-cholesterol, TG levels, of hepatic steatosis and fibrosis. Clinical studies highlighted the fact that having hypothyroidism is more common in patients with MASLD [54]. For the patients who progress to MASH, the activity of THR-β in the liver decreases in parallel with the increase of steatosis and THR-β becomes less sensitive to thyroid hormones [55].

### 2.5. MASLD and Gut Dysbiosis

Both gut and liver diseases are frequently associated with a perturbed regulation of gut–liver communication and dysbiosis. This fact, together with the disruption of the mechanical gut barrier, altered immune homeostasis and imbalanced bile salt pool, triggers enhanced gut permeability, “leaky gut”, and systemic inflammation. An altered gut microbiota (concerning its function and structure) have a great implications on the gut and liver. Researchers demonstrated that microbiota has an etiopathogenic role in gut and liver diseases, which determines the alteration of gut composition [56]. The severity of the liver dysfunction is related to the fact that liver disease disrupts gut homeostasis and determine changes in the intestinal permeability and in the composition of gut microbiota [57]. An important mediator of the gut–liver axis is represented by the gut microbiome [58].

The mechanical barrier, the immune barrier and the biological barrier represent the three major lines of defence. To maintain gut homeostasis they can interact with each other [59].

Dysbiosis can be a predisposing condition for MAFLD because the gut microbiota plays an important role in regulating metabolic homeostasis [60,61]. Recent evidence points to the important role of gut microbiota in MASLD [62].

Crucial elements in the pathophysiology of MASLD are represented by fat accumulation, hepatocyte injury, and intestinal barrier damage. The intestinal barrier is essential in absorbing nutrients and preventing the intrusion of microorganisms from the gut lumen. When the intestinal barrier function is altered, intestinal permeability increases and this in involved in the initiation and progression of intra and extrahepatic damage in MASLD. MASLD is strongly linked to gut barrier disruption, increased intestinal permeability, tight junctions alterations and dysbiosis [63].

Dysbiosis is involved in ethanol endogenous production and this directly contributes to MASLD development. At the intestinal lumen level, the undigestible carbohydrates from the diet can induce endogenous ethanol production. The endogenous ethanol arrive in the liver by the portal vein and induces liver damage which in turn aggravates MASLD pathology. Alcohol dehydrogenase and cytochrome P450 isoenzymes from the liver are involved in ethanol catabolism, consecutively to its catabolisation appear mitochondrial dysfunction which represent a causative factor for MASLD development [64].

#### 2.5.1. Mechanical Barrier Disruption in MASLD

Bacterial overgrowth in the gastrointestinal tract which can disrupt gut homeostasis and permeability can be produced by the loss of chemical substances and of mucus in the gut barrier [65].

The possibility of microbial translocation contributing to MASLD is increased by the inflammation in the intestinal mucosa and by the damage of the intestinal epithelial barrier. In MASH patients, disruption and alterations in the tight junctions has been described [66]. Tight junction proteins exhibit contractions and shift to the cytoplasm under inflammatory stimulation and conditions of hypoxia. As a consequence, the cell pores expand significantly and lead to an increased permeability of the intestinal mucosa. This, in turn, determine the translocation of intestinal bacteria and the release of bacterial byproducts (e.g., lipopolysaccharide) into the bloodstream and liver by the portal vein. As a result, liver Kupffer cells are stimulated and release inflammatory factors [65].

An important role in facilitating the entry of gut bacteria and bacterial products into the bloodstream is insured by the disruption of the gut–vascular barrier [67]. Plasmalemma vesicle-associated protein-1 (PV-1) expression, which serves as a marker of gut–vascular barrier permeability, is elevated during pathogenic events like the systemic dissemination of bacteria in MASH. During the early stages of MASH, gut–vascular barrier disruption is noticeable. By interfering with the WNT/β-catenin pathway in endothelial cells, enteric pathogens have been found to breach the gut–vascular barrier [68].

#### 2.5.2. Immunological Barrier Disruption in MASLD

The primary constituents of the immune barrier are lymphocyte- and plasma cell-secreted IgA. IgA exhibits a specific affinity for Gram-negative bacteria present in the gastrointestinal tract. However, when the intestinal mucosa is impaired, the functionality of IgA is hindered, which, in turn, causes inflammation and facilitates bacterial translocation within the intestine [69].

**Figure 2:**
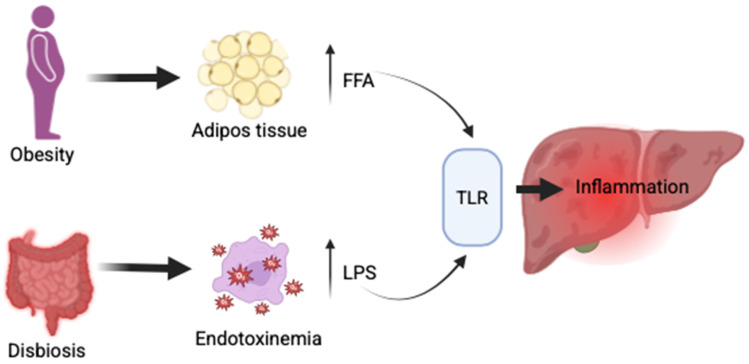
Gut–liver axis in MASLD—FFA—free fatty acids; LPS—lipopolysaccharides; TLR—toll-like receptors.

The gut–liver axis plays a critical role in the pathogenesis of MASLD. Sterile inflammation, driven by damage-associated molecular patterns (DAMPs) and pathogen-associated molecular patterns (PAMPs), significantly contributes to hepatic injury [70]. Obesity promotes the expansion of adipose tissue, leading to an increased release of free fatty acids (FFAs) into systemic circulation. Concurrently, intestinal dysbiosis alters the composition of microbiota, enhancing gut permeability and enabling bacterial overgrowth. This condition facilitates the translocation of microbial components, particularly lipopolysaccharides (LPS), into the portal vein. Both FFAs and LPS, along with other DAMPs (hyaluronic acid, fibrinogen, thermal shock proteins) and PAMPs (lipids, proteins from bacteria, viruses, fungi, parasites), activate pattern recognition receptors, especially Toll-like receptors (TLRs), on hepatic immune cells such as Kupffer cells, hepatic stellate cells, and hepatocytes [71] (Figure 2).

**Figure 3:**
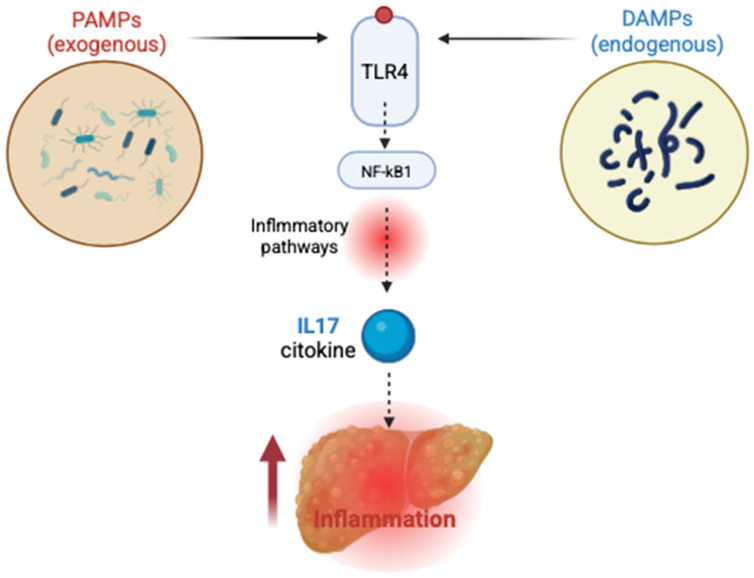
TLR4 activation in MASLD—PAMPs—pathogen-associated molecular patterns; DAMPs—damage-associated molecular patterns; TLR4, triggered by endogenous DAMPs and exogenous PAMPs, is activated and initiates downstream signaling that stimulates NF-κB and leads to cytokine production, such as IL-17, fueling a pro-inflammatory state.

The activation of toll-like receptors (TLRs), particularly TLR4, initiates complex downstream signaling cascades that involve nuclear factor-kappa B (NF-κB) and interferon regulatory factors (IRFs). These pathways ultimately lead to the transcription of pro-inflammatory cytokines, chemokines, and type I interferons. The resulting pro-inflammatory environment significantly contributes to hepatic inflammation and fibrotic remodeling. This mechanism underscores the integral role of TLR-mediated immune responses in linking metabolic dysfunction, microbial translocation, and progressive liver injury in MASLD [72] (Figure 3).

Elevated levels of serum IgA in MASLD patients were found. Thet are produced by plasma cells in secondary lymphoid organs. Both in human and mouse models of MASLD long-term inflammation and fibrosis were linked to liver-resident IgA producing cells expressing PDL1 (programmed death-ligand 1), hindering body’s capacity to avoid cancer [73]. In a mouse model of MASH, microbial factors from the gut stimulated B cells in the liver and these contributed to liver inflammation and fibrosis [74].

In the lamina propria of the gut, patients with MASLD present a decrease in FOXP3-expressing regulatory T cells (Tregs) and an increase in Th1 and CD8+T cells [75].

The mast cells are immune cells located in the intestinal barrier and are involved in releasing cytokines, histamine and proteases, which can affect the integrity of the intestinal barrier (regulating both innate and adaptive immunity). Primary proteases, trypsin and chymases, are responsible for zonula occludens-1 (ZO-1) cleavage, they increase the permeability of the gut epithelium and reduce the expression of JAM-A (junctional adhesion molecule A) [76].

An important role in the immune function at the gut barrier level is assured by the secretory immunoglobulin A (sIgA) which is produced by lymphocytes and plasma cells.

In patients with severe NASH, serum IgA levels are notably higher compared to patients in the early stages of the disease. This elevation is due to advanced fibrosis [77].

**Figure 4:**
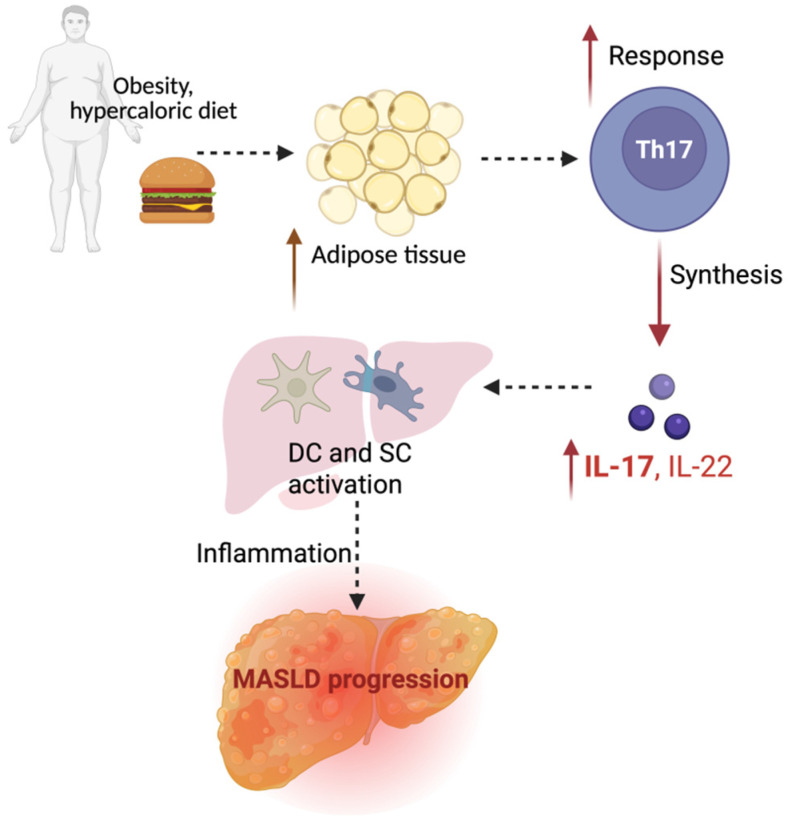
Role of IL17 in MASLD progression—Th17 cells, a subset of pro-inflammatory CD4+ T cells; IL-17 and IL-22 cytokines; DCs—dendritic cells, SCs—stellate cells in the liver.

Injured hepatocytes release DAMPs, which trigger inflammatory pathways and amplify liver damage. Among key mediators, IL-17—a pro-inflammatory cytokine mainly secreted by Th17 cells—is critically involved in linking gut-derived immune signals to hepatic inflammation. Its production, stimulated by microbial cues, contributes to steatosis, hepatocyte injury, and fibrosis. IL-17A and IL-17F, the most studied members of the IL-17 family, play pivotal roles in MASLD by promoting inflammation, disrupting metabolic balance, and driving fibrogenesis through direct activation of hepatic stellate cells [78] (Figure 4).

#### 2.5.3. Microbial Barrier Disruption in MASLD

High-fat, cholesterol, and a refined carbohydrate diet can determine gut dysbiosis and microbiome changes. It can also decrease commensal bacteria populations (involved in maintaining the integrity of the gut barrier) and increase gram-negative bacteria populations (involved in proinflammatory effect), which represent an harmful environment [79]. All these may induce an increase in gut permeability, the activation of toll-like receptors (TLRs) family and its consequent inflammation by lipopolysaccharide and endotoxin among other bacterial subproducts [80].

An important number of studies demonstrated that carbohydrate and fat diet, high-kcal diet and obesity can be involved in gut damage, gut dysbiosis, and metabolic disarrangements [81]. Other studies highlighted the fact that overweight and obese adults have less microbial gene count. Comparing lean adults with obese ones, a lower *Firmicutes/Bacteroidetes* ratio has been correlated to lean adults [82].

The gut microbiome profile of patients diagnosed with MASLD is characterized by an increased percentage of *Proteobacteria*, *Enterobacteria*, *Escherichia*, and *Bacteroides* species and a decreased percentage of *Firmicutes* species [83].

As a conclusion, a high fat content and dysbiosis can directly affect gut permeability. Also, an increase in gut inflammation can produce gut damage, dysbiosis, and an increase in gut permeability.

#### 2.5.4. Microbiome in MASLD

One of the most common effects of microbiota changes on the host is the release of bacterial metabolites that play important roles in energy homeostasis. After food is ingested, the body releases compounds named BA for digestion. BA are known to control bacterial overgrowth and maintain intestinal barrier function. Moreover, bacteria also influence BA metabolism, transforming the primary BA into secondary BA in the colon, modulating FXR and TGR5 expression, having a direct effect on glucose tolerance and homeostasis, insulin sensitivity, lipid metabolism, triglycerides (TG), and cholesterol levels, and energy expenditure by the host [63].

The close connection between the gut–liver axis and dysbiosis, one of the highlighted features in chronic liver disease, sheds light on another putative therapy for managing the disease: the restoration of intestinal microbial diversity.

## 3. MASLD Diagnosis

The diagnostic approach to MASLD primarily rules out liver pathology causes and confirms MASLD presence. Consider MASLD in individuals with: 1. Evidence of hepatic steatosis from imaging (ultrasound, CT, MRI). 2. Unexplained elevation in hepatic transaminases, notably ALT and AST. 3. Two or more metabolic risk factors, such as obesity, type 2 diabetes, dyslipidemia, or hypertension. 4. Positive family history, especially in first-degree relatives with MASLD-related cirrhosis [84].

### 3.1. Initial Clinical Evaluation

The clinical assessment begins with a detailed history and physical examination covering symptoms, comorbidities (e.g., type 2 diabetes, obesity), medication use (linked to hepatic steatosis—amiodarone, methotrexate, tamoxifen, and glucocorticoids), family history of liver disease (e.g., cirrhosis, MASLD, HCC), and alcohol intake. The physical exam should evaluate body mass index (BMI) and signs of chronic liver disease, such as hepatomegaly, jaundice, ascites, or splenomegaly. Initial labs assess liver function and metabolic status, including liver enzymes (ALT, AST, alkaline phosphatase), synthetic function markers (total bilirubin, albumin, INR), metabolic parameters (fasting blood glucose, total and HDL cholesterol, triglycerides), and complete blood count with platelets [85].

### 3.2. Imaging Assessment of Hepatic Steatosis

For patients suspected of MASLD based on imaging (e.g., transabdominal ultrasound, CT, MRI), additional imaging is unnecessary for diagnosis if other criteria are met (i.e., excluding primary etiologies and having at least one metabolic risk factor) [86]. For those without recent liver imaging (i.e., within 12 months), transabdominal ultrasound is required. In patients with MASLD, ultrasound typically shows a hyperechoic texture or bright liver due to diffuse fatty infiltration. A meta-analysis of 49 studies with 4720 patients found ultrasound sensitivity and specificity for detecting moderate to severe fatty liver were 85% and 94%, respectively, using liver biopsy as the reference standard. However, sensitivity is reduced in obese patients [87]. In a study of 187 Grade 2 obesity patients preparing for bariatric surgery, histological analysis showed hepatic steatosis in 95%, while ultrasound detection was only 49% [88].

### 3.3. Liver Biopsy: Indications and Histology

Liver biopsy is reserved for cases where noninvasive tests are inconclusive or alternative diagnoses are considered. Histologic confirmation of MASLD requires >5% steatosis in hepatocytes [89]. While imaging techniques can identify steatosis, they are generally less reliable than histology for diagnosing MASH. The use of liver biopsy for assessing inflammation and staging in all MASLD patients is limited by potential complications (like bleeding) and costs. It can clarify MASH, characterized by hepatic steatosis, hepatocyte ballooning degeneration, and lobular inflammation, typically in acinar zone 3 [90]. The NAFLD Activity Score (NAS) quantifies disease activity and guides diagnosis, especially when biopsy is required. The NAS is calculated by summing biopsy scores for steatosis (0–3), lobular inflammation (0–3), and hepatocellular ballooning (0–2), excluding fibrosis. In the study establishing NAS, scores 0–2 were generally found in cases not diagnostic for MASH. Scores of 3–4 were evenly distributed among non-diagnostic, borderline, or positive MASH cases, while 5–8 were mainly linked to diagnostic MASH cases [91]. With advancing fibrosis to cirrhosis, steatosis and inflammation may not be consistently detected. However, patients with cirrhosis and metabolic risk factors may be classified as having MASLD-related cirrhosis instead of cryptogenic cirrhosis. Evaluating MASH in patients with MASLD and another liver disease can be complex. For example, MASH patients may also have alcohol-associated liver disease, complicating the assessment of each condition via liver biopsy [92]. In a study of 3581 liver biopsies from patients with chronic liver diseases, concurrent steatohepatitis was found in 5.5% of those with hepatitis C virus (HCV) infection, including heavy alcohol users. The prevalence of steatohepatitis in other chronic liver diseases varied, from 1. 1.6% in autoimmune hepatitis to 7. 7.9% in alpha-1 antitrypsin deficiency. Importantly, none of the patients with steatohepatitis due to chronic liver disease unrelated to HCV reported heavy alcohol use [93].

### 3.4. Differential Diagnosis

The differential diagnosis of MASLD includes other conditions associated with liver steatosis, particularly in adult patients without metabolic risk factors: (1) alcohol-associated liver disease (ALD)—individuals with liver steatosis and significant alcohol consumption (>50 g daily [350 g weekly] for females and >60 g daily [420 g weekly] for males) are primarily diagnosed with alcohol-associated liver disease. A significant clinical indicator of alcohol-associated fatty liver disease is an AST to ALT ratio exceeding. (2) In contrast, in patients with MASLD, this ratio is generally <1, unless advanced fibrosis elevates AST levels [94]; (3) Chronic hepatitis C virus (HCV) infection—Liver steatosis attributable to hepatitis C genotype 3 can be ruled out with anti-HCV antibody testing [95]; (4) Wilson disease—individuals showing symptoms of Wilson disease may display elevated liver enzymes along with signs of copper overload (Kayser–Fleischer rings, neurocognitive issues) and a family history of Wilson disease; (5) parenteral nutrition—the provision of parenteral nutrition has been linked to increased liver enzymes and liver steatosis [96]; (6) drug-induced liver disease—drugs associated with liver steatosis include amiodarone, methotrexate, tamoxifen, and glucocorticoids [97]; (7) other genetic disorders—other genetic conditions such as lysosomal acid lipase deficiency (cholesterol ester storage disease), hypobetalipoproteinemia, and lipodystrophy are also part of the differential diagnosis, however, these disorders typically present in pediatric populations.

### 3.5. Staging and Fibrosis Assessment

The primary objective in staging patients with MASLD is to assess the extent of fibrosis. Typically, non-invasive imaging techniques, like Vibration-controlled Transient Elastography (VCTE) or Magnetic Resonance Elastography (MRE), are utilized. VCTE liver stiffness values: <8 kPa indicates low-risk fibrosis score (F 0-F 1), ruling out clinically significant fibrosis; 8–12 kPa is indeterminate for advanced fibrosis, where MRE may assist since ultrasound elastography is less reliable. MRE below 2.55 kPa indicates low-risk fibrosis (≤F 1), while over 3.63 kPa suggests advanced fibrosis or cirrhosis; >12 kPa indicates advanced fibrosis or cirrhosis, prompting further management and referral to hepatology for screening and preventive measures for cirrhosis complications (variceal bleeding, hepatocellular carcinoma) [98]. Combining VCTE and liver stiffness for staging MASLD patients leverages its non-invasive, point-of-care features, effectively ruling out advanced fibrosis with notable diagnostic precision. A systematic review of 44 studies revealed VCTE sensitivity of 80% and specificity of 77% in detecting advanced fibrosis (≥F 3). Research indicates MRE’s greater precision than ultrasound-based elastography for fibrosis staging, although availability and cost may limit its use [99]. A pooled analysis of 230 patients with biopsy-confirmed MASLD showed MRE’ s superior diagnostic accuracy for establishing fibrosis stages (F 0 to F 4) compared to ultrasound-based transient elastography [100]. An alternative strategy is the FIB-4 index, incorporating age, AST, ALT, and platelet count, followed by imaging (e. g., VCTE) for patients with FIB-4 ≥ 1. 1.3 [4,45,70]. A FIB-4 above 3.25 indicates cirrhosis and necessitates hepatology referral. Patients with FIB-4 over 2. 2.67 are also at risk for advanced fibrosis; thus, a hepatology referral is recommended [101].

## 4. MASLD Therapeutic Options

The following premises must be taken into account when a treatment is instituted for a patient diagnosed with MASLD:

- 90–95% of patients will not progress to liver cirrhosis;
- the progression of MASLD to liver cirrhosis lasts for 20 to 25 years;
- MASLD has the possibility to regress. When steatosis is present, regression takes place in a short time, but when fibrosis is present and adiposopathy is mitigated, regression is still possible, but takes longer [102].

When the adipose tissue capacity is surpassed by a surplus of energy, adiposopathy is present, and this represents the expression of MASLD. The adipose tissue capacity is exceeded when severe obesity is present, but can be exceeded even if the patient’s BMI is normal. This depends on the interindividual variability [103]. Adiposopathy is involved in the occurrence of MASLD, metabolic dysfunctions, cardiovascular diseases and cancer. This is the reason why in all the patients diagnosed with MASLD, the treatment targets adiposopathy. The aim is to decrease the amount of adipose tissue and to improve its function [104].

Compared to healthy subjects, patients with MASLD have an almost two-fold increase in mortality. The rate of mortality increases with the worsening of liver fibrosis and reaches up to 4-fold greater for patients with F4 fibrosis/cirrhosis [105]. The severity of fibrosis is associated not only with liver-related mortality but also with cardiovascular and cancer mortality [106]. The main causes of death are cancers, cardiovascular diseases, and only then liver diseases [105]. It seems like the obesity-associated risk for cancers is dependent on the development of MASLD [107].

### 4.1. Healthy Lifestyle and MASLD

Epidemiological research has demonstrated the link between eating habits and liver disease. Weight loss, physical activity, and a Mediterranean diet can contribute to the reduction of liver steatosis and fibrosis. A healthy lifestyle represents the primary treatment for MASLD patients [108]. The most important mortality prognostic factor in MASLD is liver fibrosis. Unfortunately, most of the patients cannot reach the target weight required to reduce liver fibrosis [109]. MASLD can also occur in lean patients with normal BMI. A normal BMI does not necessarily indicate a healthy metabolic status of the patient [110]. These patients have a poor metabolic profile compared to the healthy subjects, namely presenting hypertension, increased values of glucose, HbA1c, triglycerides, LDL-cholesterol, and decreased values of HDL-cholesterol [111].

By decreasing BMI and waist circumference, the beneficial effect of the diet can be explained. Physical activity is also involved in reducing the waist circumference [112]. For patients with MASLD, a healthy diet and regular physical activity can decrease not only cardiovascular mortality but also all-cause mortality. It is not known exactly how much physical activity should be done to protect from mortality and to have a positive impact on survival [110].

To lose weight, patients must adopt a hypocaloric diet with a 500 to 1000 kcal deficit. Epidemiological studies showed discrepant results concerning the relative importance of the quantity of energy intake over the quality of nutrients in the diet [113]. However, it is well known that high fructose consumption determines MASLD development and progression [114]. Also, the kind of fat consumed has an important role in MASLD development. A high intake of cholesterol and saturated fatty acids promotes MASLD, fibrosis, and hepatocellular carcinoma while an increased consumption of omega-3 polyunsaturated fatty acids appears to protect against the occurrence of hepatocellular carcinoma [115]. The consumption of red meat is also associated with insulin resistance and MASLD [116].

Different diets were proposed, but the most accepted one is the Mediterranean diet. The Mediterranean diet consists of a low consumption of sugars and refined carbohydrates and a high consumption of vegetables, fruits, whole grains, seeds, and nuts. Fish is allowed, but red meat is not recommended. It is basically a plant-based diet. The Mediterranean diet in MASLD patients seems to be associated with a lower risk of T2DM, cardiovascular, and cancer mortality, including from liver cancer [117].

Intermittent fasting is another proposed diet. It allows an unlimited energy intake, but the consumption is limited to certain time intervals. By maintaining this type of diet, metabolic dysfunction is improved with better glycemia and blood pressure control and a better evolution in terms of steatosis and liver fibrosis [118].

Changing dietary habits highlights the significant role of diet in the treatment of liver disease. A hypocaloric, low-fat and low-carbohydrate diet is recommended [119].

An intake of at least two coffees/day without sugar or sweeteners seems to protect against the development of hepatic steatosis/fibrosis [120], while the consumption of three coffees/day decreases the risk of developing hepatocellular carcinoma [121].

Patients with MASLD should not drink alcohol. Although there is no clear scientific evidence to indicate the prohibition of alcohol consumption in MASLD, it is known that alcohol and metabolic dysfunction are synergic involved in inducing liver disease [122]. In a recent article, it was underlined that alcohol intake can increase the risk of progression of liver disease in MASLD patients [123].

Physical activity, even when weight loss is not achieved, has a protective effect against liver steatosis, provided it is performed at least three times per week for 45 min of moderate-intensity exercise [124]. Furthermore, it was found that physical activity can improve liver stiffness and reduce hepatic steatosis, independently of dietary changes [125].

If weight loss is less than 10%, lifestyle interventions have a lower success rate. Unfortunately, not even a quarter of patients who lose weight are able to maintain their weight. Moreover, approximately 60% of them return to their initial weight in less than a year. Even if the patients reach their initial weight, the beneficial effects of the transient weight loss on metabolic dysfunction and on liver steatosis seem to persist for at least 2 years [126].

Studies reported that exercise programs help the body to counter oxidative stress, to stimulate the turnover of lymphocytes, and boost the expression of anti-inflammatory cytokines within the intestinal lining. It is proposed that exercise promotes intestinal motility, potentially leading to the shedding of loosely attached microbes from the gastrointestinal epithelium. Physical exercise fosters the proliferation of other beneficial microorganisms that play a role in maintaining a healthy mucosal immune system and gut barrier balance [127].

**Figure 5:**
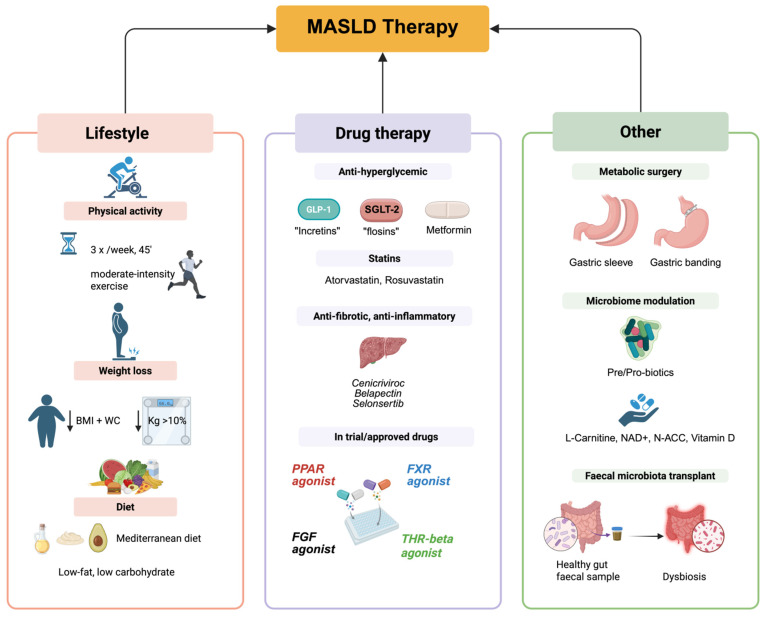
Main therapeutic approaches for MASLD. GLP-1: Glucagon-Like Peptide-1, SGLT-2: Sodium-glucose co-transporter-2, PPAR: Peroxime Proliferator-Activated Receptor, FXR: Farnesoid-X receptor, FGF: Fibroblast growth factors, THR-beta: Thyroid hormone receptor beta.

As a conclusion, the non-pharmacological approach is important, but alone is not enough in treating patients with MASLD (Figure 5) [55].

### 4.2. Drug Therapy in MASLD

Because the pathogenesis of MASLD is different, in the last decade, an increasing number of compounds have been synthesized and tested [128]. According to the development and progression of the disease, different biological targets have been identified. Special attention was granted to the histologically characterized disease pathophenotypes, ranging from steatosis to inflammation and then to fibrosis. Among the compounds tested in the last 10 years for the treatment of MASLD/MASH, very few reached the level of a phase three trial. Most of them failed to enter into clinical trials, or the studies were limited to phase one or two. Antidiabetic drugs, PPAR agonists, FXR agonists, and THR agonists are the most promising drugs in treating MASLD [31] (Figure 5).

#### 4.2.1. Anti-Hyperglycemic Drugs

For patients with steatotic liver who present insulin resistance, the use of antidiabetic drugs has shown variable results [31]. Among people with T2DM and MASH, the treatment aims to improve the aspects related to the cardiometabolic risk factors, namely glycemia, lipidemia, body weight, and blood pressure, and regarding steatohepatitis, especially if a significant fibrosis (stage ≥F2) is present. Biopsy represents the gold standard to diagnose and stage the severity of liver fibrosis, but it is an invasive method and is not feasible or acceptable for all patients. So, to establish a drug therapy, the following factors are taken into account: elevated fibrosis-4 index (FIB4) >1.3, elevated serum aminotransferase level, plasma biomarkers for liver fibrosis determination, and/or imaging such as transient elastography and magnetic resonance elastography [129].

The first anti-hyperglycemic drug to show efficacy in an early randomized clinical trial involving patients with prediabetes/T2DM and biopsy-proven MASH was *pioglitazone*, a PPAR gamma agonist that reduces insulin resistance by improving glucose utilization and lipid storage/redistribution [130]. More details concerning pioglitazone are presented below in the section PPAR agonists.

Metformin, an anti-hyperglycemic drug, affects liver fat storage and metabolism. It decreases food intake and body weight, though findings remain contradictory. Metformin inhibits gluconeogenesis, reducing liver glucose levels. After meals, it increases glucose utilization and insulin sensitivity in the liver and skeletal muscle. By inhibiting lipolysis, metformin lowers serum FFA concentrations, which are gluconeogenesis substrates. Its effects are linked to the activation of AMP-activated protein kinase (AMPK) by Peutz-Jeghers protein (LKB1), which inhibits lipogenesis and fatty acid synthesis in the liver and muscle [131].

In patients with new-onset T2DM, metformin improved the hepatic steatosis index over two years, but worsened the FIB-4 index. It enhances metabolic homeostasis in T2DM patients with steatotic liver by counteracting insulin resistance, activating AMPK, reducing ROS generation, inducing fatty acid β-oxidation, and altering gut microbiota positively [132]. Although results in treating liver steatosis are promising, metformin as monotherapy is unconvincing. Current studies focus on combining metformin with other anti-diabetic drugs like SGLT-2 inhibitors, liraglutide, and pioglitazone [133].

SGLT2 inhibitors, or “florins,” provide cardiorenal benefits as adjunctive therapy for T2DM and MASLD, improving cardiometabolic risk factors, inflammation, hepatic triglycerides, and aminotransferase levels [134].

While they significantly improve hepatic steatosis marked by MRI and non-invasive biomarkers, liver biopsy histological evaluations post-treatment remain unreported [135].

Due to their antifibrotic and antiphlogistic actions, SGLT2 inhibitors are recognized for kidney and cardio-protective effects, leading to authorization for non-diabetic conditions like kidney disease and heart failure [136]. Currently, numerous randomized controlled trials aim to demonstrate their efficacy in reducing inflammation, steatosis, and fibrosis [137].

Overall, SGLT2 inhibitors are deemed safe, with common side effects including diabetic ketoacidosis, hypotension, and genitourinary infections [138].

Among SGLT2 inhibitors, dapagliflozin and empagliflozin are the most effective. *Dapagliflozin* does not significantly affect insulin sensitivity but reduces hepatic lipid accumulation [31]. A meta-analysis of 7 trials treating patients with MASLD using dapagliflozin 10 mg showed that serum AST and ALT values decreased, insulin resistance (HOMA-IR) improved, no decreases in gamma-glutamyl transferase (GGT) were noted, and total serum cholesterol increased. No significant safety profile differences were found between groups. The DEAN phase 3 trial (NCT03723252) aims to compare dapagliflozin’s efficacy versus placebo in patients with histologically confirmed MASH, seeking improvements in liver histology score, fibrosis score, hemoglobin A1c (HbA1c), insulin resistance, and body weight within one year. *Empagliflozin* administration in T2DM patients decreases plasma liver enzyme levels and hepatic lipid accumulation, reduces the risk of diabetic ketoacidosis, and lowers extremity amputation risk, presenting a potential treatment for T2DM and MASLD patients [139].

*Glucagon-like peptide 1 (GLP-1) analogs* are effective antidiabetic medications for type 2 diabetes mellitus (T2DM). Secreted by L- and K-cells in the intestine, GLP-1 and glucose-dependent insulinotropic polypeptide (GIP) enhance insulin secretion after meals [140]. GLP-1 boosts insulin production, inhibits glucagon, and decreases appetite. Despite extensive distribution, GLP-1 receptors (GLP-1R) are not significantly present in the liver. In relation to MASLD, GLP-1 improves overall metabolism by increasing insulin sensitivity, suppressing appetite, and facilitating weight loss. The receptor agonists promote weight loss via pleiotropic actions, enhancing satiety and delaying gastric emptying [141]. Various studies indicate that GLP-1 analogs can reduce hepatic steatosis and enhance MASH, with limited results on liver fibrosis. Thus, GLP-1 analogs are promising for MASLD in T2DM and obesity. The FDA has approved seven GLP-1 receptor agonists for T2DM: exenatide, liraglutide, dulaglutide, albiglutide, lixisenatide, semaglutide, and tirzepatide. Liraglutide, semaglutide, and tirzepatide are also approved for overweight and obesity treatment [142].

In the SCALE studies, 3 mg daily *liraglutide* resulted in a 6–8% total body weight loss [143]. The LEAN study, a phase 2 trial with 52 patients comparing liraglutide to placebo, showed liraglutide significantly resolved steatohepatitis (39% vs. 9%) and slowed fibrosis progression (9% vs. 36%), though it did not improve fibrosis. The weight loss benefits did not directly correlate with histological response, indicating liver benefits may surpass weight loss effects. A few side effects, mainly gastrointestinal issues, were noted [144].

At a dose of 2.4 mg per week, *semaglutide* causes greater weight loss than liraglutide [145]. In MASLD treatment, semaglutide was included in phase 2 and 3 studies. A phase 2b study enrolled 320 patients with MASH and fibrosis (F1–F3), treated with increasing doses of semaglutide, reaching a maximum of 0.4 mg/week or placebo. The 0.4 mg dose is lower than that used for obesity treatment. MASH resolution was 40% for semaglutide versus 17% for placebo, but fibrosis showed no improvement [146]. In another phase 2 study, 71 patients with MASLD-associated cirrhosis received semaglutide 2.4 mg/week, with no improvement in fibrosis or MASH observed. There was a trend for worse fibrosis (more patients with ISHAK grade 6 and higher collagen), suggesting semaglutide may be ineffective after cirrhosis develops. This study was conducted alongside a placebo. The ESSENCE trial, a phase 3 study started in 2021, enrolled patients with MASH at F2–F3 fibrosis stages, treating them with 2.4 mg semaglutide/week and placebo. Histological and clinical endpoints will be evaluated at weeks 72 and 240, monitoring for progression to cirrhosis, hepatic decompensation, liver transplantation, and death. In the semaglutide group, side effects like gallbladder and gastrointestinal disorders, and increased lipase and amylase activity were more common [147].

*Tirzepatide* is a dual incretin, more accurately a dual GLP-1 and GIP agonist. Tirzepatide adds the beneficial effects of GLP-1 to the enhancement in adipose tissue function mediated by GIP. A phase 2 randomized clinical trial (RCT), named SYNERGY-NASH (NCT04166773), was completed at the beginning of 2024. The study assessed the efficacy and safety of tirzepatide administration in patients diagnosed with MASH. The main objective of the study was to determine whether tirzepatide leads to the resolution of MASH without worsening fibrosis. Secondary objectives included monitoring body weight, liver fat content, and changes in fibrosis stage. It was reported that approximately 75% of patients met the study’s objectives, compared to approximately 13% in the placebo group [140].

#### 4.2.2. Ketohexokinase Inhibitors

Ketohexokinase is the enzyme that transforms fructose into fructose 1-phosphate and represents the rate-limiting enzyme in fructose metabolism. Excessive fructose consumption is a risk factor for MASLD. It is associated with increased hexokinase levels, deranged fatty acid oxidation, and enhanced de novo lipogenesis. Consecutive, the hepatic steatosis and insulin signal transduction worsen [148]. The inhibition of hepatic hexokinase may decrease the fructose-induced hepatic impairement. So, in clinical trials, PF-06835919 and hexokinase inhibitors decreased hepatic lipid accumulation, but insulin resistance was not improve [149].

#### 4.2.3. Statins

MASLD is associated with hyperlipidemia. The increased transport of lipids to the liver is because lipoproteins are rich in triglycerides and cholesterol. Some small clinical trials indicated that *atorvastatin* decreases ALT serum levels and improves hepatic steatosis, and that *rosuvastatin* decreases ALT and AST serum levels and improves liver fibrosis. Statins can improve MASLD/MASH expression by reducing the risk of hepatic steatosis and fibrosis [150].

#### 4.2.4. Peroxisome Proliferator-Activator Receptor (PPAR) Agonists

The family of PPAR receptors is mostly located in the liver, brown adipose tissue, and macrophages. There are 3 types of PPAR receptors. In the liver PPARs decrease the biosynthesis of TGs, activate FFAs oxidation and increase insulin sensitivity. Older studies have demonstrated the effectiveness of PPARs in MAFLD treatment. PPAR-γ is involved in the inhibition of inflammation, in glucose and lipid metabolism regulation and in adipocyte differentiation [151].

Thiazolidinediones, commonly known as “glitazones” are potent activators of PPAR-γ and they are known as antidiabetic agents. They improve insulin sensitivity, reduce plasma FFAs level, reduce hepatic lipid accumulation and some of them seem to improve fibrosis by inhibiting the activation of hepatic stellate cells” [152].

As we mentioned above, *pioglitazone* was the first anti-hyperglycemic drug which showed efficacy in treating prediabetes/T2DM patients with MASH. In general, PPARs are dysregulated in MASLD, and the activity of *pioglitazone,* a PPAR α/γ receptor agonist, consists of steatosis improvement, hepatic biomarkers improvement, and reducing inflammation. Following the administration of pioglitazone, a histological amelioration was found by improving MASLD activity score (NAS), and no worsening of liver fibrosis was observed [153,154]. Treatment with pioglitazone is included in several guidelines as a possible treatment option for patients with T2DM and proven MASH [155]. However, the clinical use of pioglitazone is limited due to its many adverse effects, which include weight gain, fatigue, mild lower extremity edema, bone fracture in postmenopausal women, fluid retention, which can determine heart failure in patients with cardiomyopathy and bladder cancer [129]. Therefore, before pioglitazone administration, great care is required in selecting the patients [152].

*Saroglitazar* is another dual PPAR-α/γ agonist that can be used for MASLD treatment. After completing all the mandatory clinical trials, in 2020 saroglitazar was approved for MASH treatment in India, but not in other countries [156]. In the United States, saroglitazar was approved only for treating dyslipidemia in diabetic patients [157]. In a placebo-controlled RCT study (NCT03061721), a phase 2 study named EVIDENCES IV, conducted in the United States on 106 patients with MASLD or MASH, saroglitazar proved beneficial activity on the liver. At a dose of 4 mg magnesium saroglitazar, liver fat content, triglyceride, ALT, adiponectin and insulin resistance were significantly reduced [158]. Concerning the safety and tolerability, saroglitazar can cause cough and diarrhea as common adverse effects [159]. Another placebo-controlled RCT study (NCT05011305) which investigates the administration of magnesium saroglitazar to 240 patients with MASH and fibrosis is currently ongoing. The main objective of the study is to investigate the resolution of MASH without worsening fibrosis after 52 weeks of saroglitazar administration. It is also followed if saroglitazar administration improves liver fibrosis without worsening liver steatosis, liver inflammation or ballooning [160].

*Lanifibranor* is a pan-PPAR agonist which acts as an agonist on all three PPAR isoforms because it can bind to different regions of the PPAR α-, δ-, and γ-ligand domain [161]. This is why in MASLD patients, lanifibranor can have a better activity in reducing inflammation, liver fibrosis and metabolic risk factors compared with a single PPAR agonist [161]. In a phase 2b placebo-controlled RCT (NCT03008070) study, named NATIVE study, in which 247 participants were enrolled, the activity of lanifibranor (administered for 24 weeks) was compared with placebo. It demonstrated a higher activity in reducing the SAF-A score (Steatosis Activity Fibrosis score) by at least two points without deterioration of fibrosis [162]. Due to the fact that promising results were obtained, a phase 3 placebo-controlled RCT (NCT04849728) study, named the NATIV3 study was started. The study is still ongoing and is estimated to be completed in October 2026. This study aims to enroll 1000 patients diagnosed with active MASH and liver fibrosis (stage F2/F3) and to measure the resolution of MASH with improvement in fibrosis at week 72. Concerning the side effects, they are more frequent in the lanfibranor group compared to the placebo group and consist of nausea, diarrhea, peripheral edema, weight gain, and anemia. The positive results of this study could lead lanifibranor to a step of approval [23].

#### 4.2.5. Farnesoid-X Receptor (FXR) Agonists

FXR is a nuclear receptor activated by BA, mainly in the liver but also in the small bowel. It modulates bile acids, cholesterol, glucose metabolism, lipogenesis, inflammation, fibrogenesis, intestinal integrity, and vascular remodeling [163]. A complex pathway governs BA biosynthesis. Luminal BAs reabsorb into the ileal enterocyte, interacting with nuclear FXR to upregulate FGF-19 expression. During enterohepatic circulation, FGF-19 binds hepatic FGFR4/β-Klotho, inhibiting CYP7A1 expression, the rate-limiting enzyme in BA synthesis [47]. This prevents excessive bile acid accumulation in the liver. Liver FXR activation improves glucose tolerance by increasing glycogenogenesis and decreasing gluconeogenesis [164]. Additionally, it reduces hepatic fat accumulation through small heterodimer partner expression and CYP7A1 activity [165], while upregulating FGF-19 and FGF-21, which have anti-steatogenic and anti-fibrotic effects [166].

An analog of the primary BA chenodeoxycholic acid, named *obeticholic acid* (a semi-synthetic BA), was tested for a potent agonistic activity on FXR and for its possible beneficial metabolic effects on glucose and hepatic lipid metabolism. In order to determine the long-term effects of obeticholic acid on MASH and fibrosis in patients with stage 1–3 fibrosis, a large phase 3 randomized, placebo-controlled trial was performed. Unfortunately, obeticholic acid treatment did not correspond in terms of MASH resolution and side effects. For about 10% of cases, the treatment was discontinued due to the presence of a dose-dependent pruritus, and about 50% of patients developed hypercholesterolemia requiring newly prescribed statins [167].

Other synthetic FXR agonists are being tested and might produce fewer adverse events than obeticholic acid. *MET642* is included in a phase 2 clinical trial enrolling MASH patients (NCT0477396). *MET409* alone, in a phase 1b trial, decreases the amount of lipids in the liver, and currently is associated with empagliflozin in a phase 2b trial. The non-BA *tropifexor* is another highly potent FXR agonist tested in a phase 2 clinical trial (NCT02855164), a study named FLIGHT FXR. In the study, MASH patients diagnosed with fibrosis stage between 1 and 3 were enrolled. Compared to placebo, tropifexor decreases the amount of lipids in the liver, reduces body weight and transaminases [168].

#### 4.2.6. Fibroblast Growth Factors (FGF) Agonists

The FGF superfamily includes FGF19 and FGF21, which have beneficial effects on glucose and lipid metabolism. In animal models, the administration of FGF19 and FGF21 decreases body weight and lipid mass and improves insulin sensitivity, hepatic steatosis, and serum lipid levels. A potential explanation may be the reduced expression of genes involved in TG biosynthesis or the inhibition of SREBP1 [169].

FGF-19 is a gut hormone induced by the activation of FXR. It is downregulated in patients with MASH and has a beneficial activity in maintaining BA, carbohydrates, and energy homeostasis. However, it is associated with a bad prognosis because it has a potential carcinogenic effect, being involved in promoting hepatocellular carcinoma [170].

*Aldafermin* (NGM282) is an engineered analog of recombinant human FGF19 with a 95.4% homology, made up of 190 amino acids. In animal models of MASH, it showed beneficial histological effects at the liver level [171]. Aldafermin can inhibit BA synthesis and regulate metabolic homeostasis.

In a 24-week phase 2b trial carried out on patients with MASH-related stage 2 or 3 fibrosis, aldafermin did not improve liver fibrosis but decreased hepatic lipid accumulation by 7.7% [172]. In another phase 2b trial aldafermin was well tolerated, without a significant dose-dependent response in fibrosis [173].

The activity of FGF-21 in the liver is to inhibit “de novo” lipogenesis, stimulate FFA oxidation, incorporate TG into VLDL and deliver them into the blood. In addition, FGF21 can modulate obesity and hepatic metabolic homeostasis via increased energy consumption and insulin sensitivity. The secretion of FGF21 is dependent on starvation, nutritional stress, a high-fat diet, or a nutritional restriction diet [174]. The human FGF-21 has a short half-life and thus to obtain a structural FGF-21 analog, modifications are required in the aim to increase both its half-life and its stability [175].

*Pegozafermin (BIO89-100)*, a pegylated FGF-21 agonist, was developed for treating severe hypertriglyceridemia and MASH by subcutaneous administration. It must be administered once at 14 days, because it has a longer half-life, due to the fact that it is pegylated [176]. In a phase 2b placebo-controlled RCT (NCT04929483) study, pegozafermin improved fibrosis in 222 patients with MASH [177]. Due to this fact, in March 2024, pegozafermin entered a phase 3 clinical trial (NCT06318169) to determine not only the efficacy of pegozafermin in patients diagnosed with MASH and fibrosis but also to evaluate its safety. The study is expected to end in 2029 [178].

*Efruxifermin* consists of a human IgG1-Fc domain and two altered FGF-21 and is a fusion protein with a great stability in the body. It represents another drug from this group which has good potential in treating MASLD [179]. In 2023, the first results of the placebo-controlled phase 2b RCT (NCT04767529), named HARMONY, were published, which enrolled patients with MASH and F2 or F3 fibrosis stage. The main objective of the study was to find an improvement in at least one fibrosis stage, without worsening of MASH, after the administration of 28 mg/50 mg efruxifermin for 24 weeks. The study concluded that efruxifermin improves liver fibrosis, more accurate for the group that received 28 mg efruxifermin an improvement of 36% was achieved compared to 19% for the placebo group and for the group that received 50 mg efruxifermin an improvement of 33% was achieved compared to 19% for placebo group [180]. In late 2023, efruxifermin entered two ongoing placebo-controlled phase 3 RCT studies. SYNCHRONY (NCT06215716) is the first study, in which are expected to be enrolled 1000 participants and which has as main objective to find an improvement in at least one fibrosis stage after 52 weeks of treatment with efruxifermin. It is a histology study and it is expected to end by March 2027. SYNCHRONY Real-World (NCT06161571) represents the second RCT study, which investigates the tolerability and the safety of efruxifermin. The study aims to enroll 700 patients diagnosed with MASLD and it is expected to end until October 2026 [181].

#### 4.2.7. Thyroid Hormone Receptor Beta (THR-β) Agonists and MASLD

Selective THR-β agonists improve the conversion of T4 to T3 and likely enhance mitochondrial function, in addition to the anticipated beneficial metabolic effects [52].

*Resmetirom* (trade name Rezdiffra) was very quickly approved by the US Food and Drug Administration in March 2024, to improve liver condition. It became the first drug approved for treating non-cirrhotic MASH in adults diagnosed with F2 to F3 fibrosis stages [182]. Resmetirom is orally administered and compared with the T3 hormone. Resmetirom is about 28 times more selective for THR-β than for THR-α. Also, it reaches the extrahepatic tissues in a very small amount [183]. After oral administration, Resmetirom improves liver fat content and liver fibrosis. Liver enzymes, lipid metabolism parameters, and inflammatory biomarkers are also improved without major safety concerns. However, nausea and diarrhea have been reported as side effects [172].

*VK2809* represents another THR-β agonist that targets the liver and can be orally administered. Two double-blind RCTs evaluated its efficacy and safety. One is a phase 2a study (NCT02927184), a 12-week study, in which 59 patients with MASLD and hypercholesterolemia were enrolled, which was completed in 2019. It showed a significant reduction in LDL-cholesterol, lipoprotein A, apolipoprotein B, and ALT levels. The other, a phase 2b study (VOYAGE, NCT04173065), a 52-week study, began in May 2023, was completed in 2024, and was carried out on patients with MASH and biopsy-proven fibrosis. The results indicated a decrease in the hepatic fat content, so the main objective of the study was met. Both studies reported mild side effects, which indicates that *VK2809* has a good safety profile [184]. VK2809 is still under investigation but shows good potential [185].

#### 4.2.8. Anti-Fibrotic and Anti-Inflammatory Agents

*Cenicriviroc* is an antagonist compound, administered orally, which blocks chemokine 2 and 5 receptors. This two receptors are involved in the fibrosis and the inflammation of the liver. In the CENTAUR phase 2b trial, cenicriviroc ameliorated liver fibrosis without worsening MASH, but did not achieve any histological improvement in NASH. The AURORA phase 3 trial (NCT03028740) was prematurely interrupted because the interim analysis prove no efficacy for cenicriviroc [186].

In the TANDEM phase 2b trial (NCT03517540) the combined treatment of cenicriviroc with tropifexor was studied. Was reported that in patients with biopsy proven MASH with fibrosis, the association was safe and it decreased body weight and ALT. Unfortunately, when considering the histological evaluation criteria, the combination of cenicriviroc plus tropifexor did not prove a was superior activity compared to monotherapy with cenicriviroc or tropifexor [187].

Galectin-3 is a β-galactoside-binding lectin involved in fibrosis and inflammatory response. In a phase 2b NASH-CX clinical trial (NCT02462967), *belapectin* (a complex carbohydrate) targeted galectin-3 but did not present a significant activity on fibrosis and inflammation when compared with placebo. In patients diagnosed with MASH-related cirrhosis without esophageal varices, belapectin decreased the hepatic venous pressure gradient. A phase 2b/3 trial (NCT04365868) named NAVIGATE will assess for 18 months the administration of belapectin compared to placebo, in MASH patients with compensated cirrhosis, to monitor those who will develop new esophageal varices and clinically significant cirrhosis-related events [188].

*Selonsertib* is an inhibitor of apoptosis signal-regulating kinase-1 (ASK1). The phase 3 trials named STELLAR 3 and 4 showed that the oral administration of selonsertib to patients diagnosed with stage 3 fibrosis or compensated cirrhosis was well tolerated. Still, they did not improve the fibrosis without worsening MASH [189].

Other compounds are tested for their anti-inflammatory and anti-fibrotic activities. For example, GB1211 targets galectin 3, DFV890 targets nucleotide oligomerisation domain (NOD)-like receptor family pyrin domain-containing 3 (NLPR-3), nimacimab targets cannabinoid 1 receptor (CB1) tested in phase 1 trials, trialstipelukast (a leukotriene), or nitazoxanide (an antiparasitic agent) tested in phase 2 trials. Results about safety and efficacy are therefore awaited [31].

#### 4.2.9. Microbiome Modulation in MASLD

Preclinical and clinical studies suggest that the microbiome could be a novel target for alleviating the pathophysiology of MASLD [63].

To improve the imbalanced microbiota of the host the use of probiotics is recommended. Probiotics are a group of beneficial microorganisms that actively colonizes the human gut. Probiotic supplementation decreases the presence of pathogenic bacteria and reduces the production and entry of harmful compounds into the liver [190].

The probiotic beneficial effect may result from a combination of actions, which may be related to the enzymes or metabolites produced by specific strains.

Concerning the function of the intestinal barrier, studies underlined the fact that probiotics stimulate immune function against enteric pathogens and regulates intestinal inflammation by PPARγ expression. Moreover, probiotics improve the function of the intestinal barrier by altering the characteristics and secretion of intestinal mucus not only by the suppression of the host’s inflammatory response. The composition of intestinal microorganisms is also involved in changes in the nature of the mucus and in increasing its secretion. The expression and localization of tight junction proteins and genes related to mucin production is promoted by probiotics [191].

Prebiotics are indigestible food ingredients that are involved in improving the host’s health by stimulating selectively the activity and growth of specific bacterial colonies. They function by increasing the activity of probiotics and have a positive impact on the human body. More efficacy presents the use of symbiotics (the combination of probiotics with prebiotics) [192].

Correction of gut dysbiosis can combat the hyperpermeability, the disrupted intestinal barrier, the flow of bacterial products (i.e., lipopolysaccharides), the immune system and inflammatory activation in the intestine and the endothelial barrier in the liver and at systemic level. The close relation between overweight/obesity and the steatotic liver opens up new avenues of approach targeting gut microbiota [60]. For example, a double-blind, randomized placebo-controlled pilot study which included 26 obese patients was conducted over a period of 6 months. The patients received a symbiotic multispecies daily containing 1.5 × 10^10^ CFU of a blend of *B. bifidum W23*, *B. lactis W51*, *B. lactis W52*, *L. acidophilus W37*, *L. casei W56*, *L. brevis W63*, *L. salivarius W24*, *Lc. lactis W58*, *Lc. lactis W19*, glucomannan galacto-, fructo-oligosaccharides, minerals, and B_2_ and D_3_ vitamins. Beneficial effects on hip circumference was obtained and overall quality of life was improved [193].

A promising treatment against MASLD consists in a mixture of metabolic cofactors composed of L-carnitine, nicotinamide riboside, N-acetyl cysteine and betaine [194]. Vitamin D supplementation has been reported to improve insulin resistance in MASLD [195] and omega-3 FFAs can be helpful in MASLD [9]. A multi-ingredient supplementation improves pathological MASLD features in the liver, reduces inflammation and steatosis [196].

Although the approval on the market of resmetirom for MASLD treatment represents a great success, nowadays it is considered that the combination of some suitable drugs could be much more beneficial in treating MASLD with reduced adverse effects [197].

Since research in the field has intensified and many compounds are in the final stages of the study, new drugs for MASLD treatment are expected to be soon introduced onto the market [198].

### 4.3. Metabolic Surgery

#### 4.3.1. Bariatric Surgery

Bariatric surgery can be performed on patients diagnosed with MASLD, but it is not primarily performed for MASLD. It can be used to correct the severe obesity-related comorbidities in patients with MASLD [31].

Two types of intervention are known when talking about bariatric surgery. One method is only restrictive, namely, by decreasing the stomach capacity the calory intake is also restricted. Sleeve gastrectomy and adjustable gastric banding represent examples of restrictive bariatric surgery. The other method bypasses the proximal small bowel, which involves a decrease in its absorptive surface. This method is not only restrictive it is also malabsorptive. The Roux-en-Y gastric bypass and biliopancreatic diversion with duodenal switch represent examples of restrictive and malabsorptive bariatric surgeries. Compared to malabsorptive methods, restrictive methods cause less weight loss but produce fewer adverse effects [199]. Different results are obtained in the treatment of MASLD depending on the type of intervention performed. Thus, it was found that the adjustable gastric banding is less effective in improving liver histology, while the gastric bypass and sleeve gastrectomy have similar effects [200].

The beneficial effects of bariatric surgery in MASLD treatment are dependent on weight loss, and seem to last for approximately five years. Several studies have shown that bariatric surgery promotes steatosis resolution, MASH resolution without worsening fibrosis in about 80% of the cases, and fibrosis regression in about 70% of the cases. Even if initially the fibrosis is advanced, for more than 50% of cases a complete fibrosis resolution may be achieved [201]. In an open-label trial, 300 patients diagnosed with MASH were randomized to receive lifestyle intervention or bariatric surgery. The study proved that bariatric surgery was associated with a 70% higher chance of achieving fibrosis improvement of at least one stage and with a 50% decreased risk of worsening fibrosis [202]. In approximately 75% of the patients with steatohepatitis, there was an improvement in histological characteristics, namely in ballooning and lobular inflammation [203]. It is essential to highlight that bariatric surgery in MASLD patients is associated with a decreased risk of major liver outcomes and of cardiovascular events [204].

In the case of patients with liver cirrhosis, bariatric surgery must be proposed with great caution because mortality rate exceed up to 1%. For the patients diagnosed with decompensated cirrhosis, bariatric surgery is contraindicated because the mortality rate reaches up to 20% [205]. In patients with MASH, the complication rate of bariatric surgery does not increase [206].

#### 4.3.2. Endoscopic Bariatric Interventions

Most of the studies carried out in the past indicate that the beneficial effects of endoscopic bariatric interventions in the treatment of MASLD patients are low. Endoscopic bariatric interventions are available, such as aspiration therapy, balloons, endoscopic sleeve gastroplasty, which causes the restriction of food intake, and endobarrier and duodenal mucosal resurfacing, which represent small bowel devices that target insulin resistance and metabolic profiles. A recent meta-analysis, which included 863 patients diagnosed with MASH, suggested that endoscopic bariatric interventions may determine a histologic improvement [207]. Due to the fact that after balloon removal, most patients regain weight, the intragastric balloon seems to have only a transient effect on weight and therefore is not suitable for long-term therapy [208]. Regarding endoscopic sleeve gastroplasty, a two-year follow-up study was carried out on obese MASLD patients treated in this way. A sustained weight loss and an improvement in liver histology were registered. Namely, for 20% of the patients that presented baseline F3–F4 fibrosis, it regressed up to F0–F2 fibrosis [209].

### 4.4. Faecal Microbiota Transplant

Gut microbiota is associated with metabolic diseases, including obesity, insulin resistance, and MASLD. Concerning the application of faecal microbiota transplant as a treatment, single or combined, for MASLD, some studies on animals show that the total faecal microbiota transplant from healthy donors to high-fat diet (HFD) animals improved gut permeability and decreased inflammation and steatosis in the liver of recipients [210].

In clinical practice, a faecal microbiota transplant is approved only for the treatment of the recurrent infection with *Clostridium difficile* [211]. However, some randomized clinical trials were developed that propose a faecal microbiota transplant to improve obesity, metabolic syndrome, and MASLD [212]. The reduced sample size and the heterogeneity of donors and recipients represent the limitations of such studies. Despite significant changes in bacterial populations, it is hard to find remarkable improvements in MASLD pathophysiology when a faecal microbiota transplant is used as a single treatment. Most clinical trials show a tendency for body weight and adiposity reduction in long-term faecal microbiota transplant applications. Regarding the effects of a faecal microbiota transplant on the liver, fat accumulation in the liver was decreased. In line with this, the faecal microbiota transplant has been proposed as a treatment for MASLD [213].

## 5. Future Perspectives

In the future, with the aim of ensuring a more effective MASLD treatment, is recommended to subcategorize MASLD depending on the severity of a patient’s metabolic syndrome symptoms. Perhaps the classification of MASLD according to the degree of fibrosis will lose its usefulness [214].

Combination therapy targeting different pathological pathways and personalized treatment is needed [109]. For this purpose, several aspects in the treatment of MASLD should be clarified, namely, the use of monotherapy must be clearly defined, the dose and the safety of such combination must be rigorously tested and clinical trials must be carried out on a large number of patients [215]. Furthermore, more researches are needed to understand the intricacies of miRNAs function and develop effective miRNA-based therapies for MASLD.

## 6. Conclusions

MASLD is a liver disease with a great impact on patients’ global health. The establishment of a healthy lifestyle is essential but not sufficient. Therefore, other treatment options are needed. Taking into account the fact that MASLD is a multisystemic disease, a multidisciplinary approach is required. This is the reason why adopting a healthy lifestyle together with a monotherapy may not give results. To obtain good results in treating MASLD, a combination therapy that can target different pathological pathways and organ systems must be studied intensively. A personalized therapy should be instituted for patients who do not respond to conventional treatment. Taking into account the increasing prevalence of MASLD and the fact that the age at which it is diagnosed is decreasing, regular medical check-ups are recommended among the population, to diagnose the disease in its initial stages and before complications appear.

## Abbreviations

ALD	alcohol-associated liver disease
ALT	alanine aminotransferase
AMPK	AMP-activated protein kinase
ApoB	apolipoprotein B
ARFI	acoustic radiation force impulse
ASK1	apoptosis signal-regulating kinase-1
AST	aspartate aminotransferase
BA	bile acids
BMI	body mass index
cAMP	cyclic adenosine monophosphate
CB1	cannabinoid 1 receptor
CT	computed tomography
DAMPs	damage-associated molecular patterns
DCs	dendritic cells
EVs	extracellular vesicles
FFAs	fatty acids
FGF15/19/21	fibroblast growth factor 15/19/21
FIB4	fibrosis-4 index
FoxO1	forkhead box protein O1
FXR	farnesoid X receptor
GGT	gamma-glutamyl transferase
GIP	glucose-dependent insulinotropic polypeptide
GLP-1	glucagon-like peptide-1
HbA1c	hemoglobin A1c
HCC	hepatocellular carcinoma
HCV	hepatitis C virus
HDAC	histone deacetylase
HFD	high-fat diet
HK2	hexokinase 2
HOMA-IR	homeostatic model assessment of insulin resistance
IL-6/17/22	interleukine-6/17/22
INR	international normalized ratio
JAM-A	junctional adhesion molecule A
LDL	low-density lipoprotein
LPS	lipopolysacharides
LXR	liver X receptor
MAFL	metabolic-associated fatty liver
MASH	metabolic dysfunction-associated steatohepatitis
MASLD	metabolic dysfunction-associated steatotic liver disease
mGPD	mitochondrial glycerophosphate dehydrogenase
MRI	magnetic resonance imaging
MRE	magnetic resonance elastography
MTTP	microsomal triglyceride transport protein
NAFL	nonalcoholic fatty liver
NAFLD	nonalcoholic fatty liver disease
NAS	NAFLD activity score
NASH	non-alcoholic steatohepatitis
NF-kb	nuclear transcription factor Kappa B
NLPR-3	family pyrin domain-containing 3
NOD	nucleotide oligomerisation domain
NR1A2	nuclear receptor subfamily 1, group A, member 2 = TR-β
PAMPs	pathogen-associated molecular patterns
PDL1	programmed death-ligand 1
p-HSL	phosphohormone-sensitive lipase
PKM2	pyruvate kinase isozyme type M2
PPAR	peroxisome proliferator-activator receptor
p-PLIN1	phospho-perilipin 1
PV-1	plasmalemma vesicle-associated protein-1
RCT	randomized clinical trial
ROS	reactive oxygen species
SAF-A score	steatosis activity fibrosis score
SCs	stellate cells
SFL	simple fatty liver
SGLT-2	sodium-glucose cotransporter 2
sIgA	secretory immunoglobulin A
SREBP	sterol regulatory element-binding protein
T3	triiodothyronine
T4	thyroxine
T2DM	type 2 diabetes mellitus
TG	tryglycerides
TGF-β	transforming growth factor-β
THR (α and β)	thyroid hormone receptor isoforms (α and β)
TLRs	toll-like receptors
Tregs	regulatory T cells
TSH	thyroid-stimulating hormone
VCTE	vibration-controlled transient elastography
VLDL	very low-density lipoprotein
ZO-1	zonula occludens-1
